# Uso de Realidad Virtual Basada en Actividades de la Vida Diaria en la Rehabilitación Cognitiva del Ictus: Una Revisión Sistemática

**DOI:** 10.31083/RN37507

**Published:** 2025-04-28

**Authors:** Florencia Sofía López-Isola, Daniel Íncera-Fernández

**Affiliations:** ^1^Facultad de Ciencias de la Salud, Universidad Alfonso X el Sabio, 28016 Madrid, España; ^2^Facultad de Ciencias de la Salud, Universidad Internacional de Empresa, 28023 Madrid, España

**Keywords:** realidad virtual, accidente cerebrovascular, actividades cotidianas, rehabilitación de accidente cerebrovascular, neuropsicología, virtual reality, stroke, activities of daily living, stroke rehabilitation, neuropsychology

## Abstract

**Introducción:**

La realidad virtual (RV) genera un ambiente virtual en el que se puede interactuar como si fuese real. En personas con Ictus, que representa la segunda causa de discapacidad en Europa, se ha estudiado el uso de RV en rehabilitación cognitiva mediante diferentes tipos de tareas, que podrían tener diferente impacto. Por esta razón, el objetivo de este trabajo es revisar el uso de tareas de RV basadas exclusivamente en actividades diarias para la rehabilitación cognitiva de personas mayores de 18 años que hayan padecido Ictus.

**Método:**

Se realizó una búsqueda en *PubMed*, *Web of Science *y *Scopus*, obteniéndose 531 artículos que, luego de aplicar los criterios de inclusión/exclusión, se redujo a ocho (seis ensayos clínicos aleatorizados y dos estudios cuasiexperimentales).

**Resultados:**

El mayor número de resultados positivos se obtuvo en el funcionamiento cognitivo global pero en funciones cognitivas específicas fueron minoría.

**Conclusiones:**

Los resultados destacan la necesidad de la realización de un mayor número de estudios y con mayores muestras, para obtener resultados más robustos. Esta revisión pone foco en la importancia de seguir estudiando este tema debido a la posibilidad de interesantes líneas de investigación futura.

## Abstract (en)

**Background:**

Virtual reality (VR) generates a virtual environment with which one can interact as if it were real. In Stroke, which represents one of the highest causes of cognitive impairment in Europe, the use of VR in cognitive rehabilitation has been studied through the performance of different types of tasks, which could have different impacts. For this reason, the objective of this study was to review the use of VR tasks based exclusively on daily activities for the cognitive rehabilitation of people with stroke over 18 years of age.

**Methods:**

A search was carried out of the databases PubMed, Web of Science, and Scopus, obtaining 531 articles that, after applying inclusion/exclusion criteria, were reduced to eight (six randomized clinical trials and two quasi-experimental studies).

**Results:**

The number of positive results was higher than the number of negative results only in global cognitive function. In specific cognitive functions the number of positive results was lower than the negatives.

**Conclusions:**

These results highlight the need to carry out more studies with larger samples to obtain robust results and conclusions. Furthermore, this study highlights the value of research in this topic due to the interesting lines of future research.

## 1. Introducción

### 1.1 Ictus

El Ictus es un tipo de daño cerebral adquirido (DCA), el cual se define como una lesión o lesiones que ocurren en el cerebro alterando su funcionamiento normal [1]. En Europa el Ictus es la segunda causa de muerte y la primera causa de discapacidad [2]. Esta discapacidad es consecuencia de las secuelas que ocasionan esas lesiones, ya que pueden afectar a distintas áreas, como el área motora, el lenguaje, el funcionamiento cognitivo, la conducta y el área sensorial [3, 4]. Esta afectación tendrá gran impacto en las actividades de la vida diaria (AVD) [5] y su nivel de gravedad dependerá de distintas variables como la causa del Ictus (hemorrágica o isquémica) y la severidad y ubicación de la lesión [4].

La intervención en este ámbito debe ser individualizada y abordada de manera holística con el objetivo de que la persona recupere el mejor nivel posible en las áreas afectadas [3, 6].

### 1.2 Rehabilitación Cognitiva

La rehabilitación cognitiva se define como el conjunto de actividades terapéuticas orientadas a la funcionalidad, basadas en la evaluación y entendimiento de los déficits específicos del paciente [7]. Tradicionalmente, la rehabilitación cognitiva se ha llevado a cabo mediante un enfoque dónde el objetivo era mejorar la alteración cognitiva mediante ejercicios descontextualizados (p. ej., tareas de cancelación, búsqueda de similitudes). Sin embargo, debido al debate generado sobre su efectividad, surge una perspectiva contextualizada. Esta tiene como cometido establecer objetivos funcionales relevantes para la persona y promocionar una mayor participación en las AVD [8]. Actualmente, las recomendaciones de consenso incluyen ambos tipos de tareas, pero se sustentan principalmente en esta última perspectiva [9, 10, 11, 12]. La intervención desde la perspectiva contextualizada puede llevarse a cabo tanto en una consulta, como en contextos diarios [8]. Sin embargo, este último aspecto no siempre es fácil de implementar debido a razones de seguridad, características de los usuarios, o escasez de recursos [13]. En este sentido, los sistemas de realidad virtual pueden ser una alternativa frente a estas limitaciones, ya que en ellos se logra representar entornos similares a los encontrados en la vida real.

### 1.3 Realidad Virtual

El término realidad virtual (RV) se define como “un ambiente digital generado por computadora en el que se puede experimentar e interactuar como si ese ambiente fuera real” ([14], p.8). Es posible representar este ambiente a través de distintos dispositivos que pueden clasificarse según su nivel de inmersión (capacidad del dispositivo para aislar al usuario de su entorno físico real), en inmersivos (p. ej., gafas de RV), semi-inmersivos (p. ej., proyecciones en pantallas cóncavas) o no inmersivos (p. ej., pantallas planas como ordenadores) [15, 16, 17]. Estos sistemas presentan importantes ventajas para su uso en neurorrehabilitación física y cognitiva. Esto es debido a características como el ajuste de la dificultad de las tareas, la presencia de *feedback* inmediato y el aumento de la motivación de los participantes debido a sus características lúdicas [18].

### 1.4 Realidad Virtual en Rehabilitación del Ictus

La RV se ha empleado en diversos estudios para la rehabilitación cognitiva en Ictus [19, 20, 21, 22]. Las tareas de rehabilitación presentadas en los programas de RV han sido diversas y pueden agruparse principalmente en dos categorías: tareas contextualizadas o basadas en actividades diarias (p. ej., cocinar, comprar, hacer recados) y tareas descontextualizadas. La mayoría de las revisiones sistemáticas realizadas sobre el uso de RV en rehabilitación cognitiva del DCA, abarcan estudios que emplean RV que presenta ambos tipos de tareas [23, 24, 25]. La guía del *International Group of Cognitive Research and Clinicians* (INCOG 2.0), si bien establece un nivel de evidencia alto para el uso de RV en rehabilitación de funciones ejecutivas (FE), destaca una necesidad de profundizar en las características ideales de la RV y sus programas [9]. Hasta la fecha, sólo una revisión ha realizado la diferenciación entre tipos de tareas presentadas en la RV, incluyendo exclusivamente estudios sobre tareas de RV basadas en actividades diarias [26]. Sin embargo, se llevó a cabo revisando la efectividad de su uso en personas con DCA de cualquier edad, tanto en intervención como en evaluación de secuelas cognitivas y también motoras.

Hasta el momento, no se ha revisado el impacto de tareas de RV exclusivamente basadas en actividades diarias y sólo en el ámbito de rehabilitación cognitiva de personas con Ictus mayores de 18 años. Por ello, y acompañado al rápido crecimiento científico en esta área, se considera la realización de una revisión sistemática sobre el tema.

A partir de lo expuesto, se plantea como objetivo general:

1. Analizar la evidencia disponible sobre el uso de la RV basada en actividades diarias en la rehabilitación cognitiva de personas mayores de 18 años que hayan padecido Ictus.

Como objetivos específicos:

1. Analizar si tras la intervención de RV basada en actividades diarias en personas que hayan padecido Ictus existe evidencia de mejora en las medidas del funcionamiento cognitivo global, FE, atención o memoria.

2. Identificar si tras la intervención de RV basada en actividades diarias en personas que hayan padecido Ictus alguna de las áreas cognitivas evaluadas muestra una mejora de mayor tamaño frente a las demás.

3. Detectar qué instrumentos de medida se utilizaron para la evaluación del funcionamiento cognitivo en los estudios revisados.

4. Comprobar la existencia de medidas de seguimiento en los estudios revisados y describir su resultado.

Como hipótesis:

1. Tras la intervención de RV basada en actividades diarias en personas que hayan padecido Ictus, mejorarán las medidas de resultados del funcionamiento cognitivo global, FE, atención y memoria.

2. Las mejoras después de la intervención de RV basadas en actividades diarias en personas que hayan padecido Ictus, se darán especialmente en el dominio de FE.

## 2. Método

Para llevar a cabo esta revisión sistemática se siguieron las pautas de la declaración *Preferred Reporting Items for Systematic reviews and Meta-Analyses* (PRISMA) [27] (**Material Suplementario-PRISMA checklist**). Siguiendo el objetivo principal establecido, se conformó una pregunta *population, intervention, outcome* (PIO) para guiar la búsqueda (ver Tabla 1).

**Tabla 1.** **Pregunta PIO**.

Population	Personas con Ictus mayores de 18 años
Intervention	RV con tareas basadas en actividades diarias
Outcome	Medidas del funcionamiento cognitivo global, funciones ejecutivas, atención o memoria
Pregunta PIO	En personas con Ictus mayores de 18 años, ¿Qué impacto tiene la RV basada en actividades diarias en el funcionamiento cognitivo?

PIO, *population, intervention, outcome*; RV, realidad virtual.

### 2.1 Búsqueda Bibliográfica

La búsqueda se realizó en las bases de datos *Pubmed*, *Web of science *y* Scopus*. Además, se analizaron las referencias de los artículos seleccionados para identificar estudios que no se hubieran localizado inicialmente. A continuación, se presentan los términos utilizados combinados mediante los conectores booleanos “*AND*” y “*OR*”: “*acquired brain injury*”, “*stroke*”; “*virtual reality*”, “*virtual environment*”; “*cognitive rehabilitation*”, “*cognition*”, “*cognitive functions*” (ver Tabla 2). Los rastreos de artículos fueron realizados en la categoría *Title/Abstract*. La búsqueda inicial fue llevada a cabo en el mes de diciembre de 2023.

**Tabla 2.** **Estrategia de búsqueda empleada**.

*Population*	*Intervention*	*Outcome*
*Acquired brain injury*	*Virtual reality*	*Cognitive rehabilitation*
*Stroke*	*Virtual environment*	*Cognition*
		*Cognitive functions*

Para la selección de los estudios se siguieron los siguientes criterios de inclusión:

1. Participantes que hayan padecido Ictus (en fase aguda o crónica) mayores de 18 años.

2. Sistemas de RV de cualquier nivel de inmersión que representaran tareas de la vida diaria (al menos un 75% sobre actividades diarias).

3. Ensayos clínicos aleatorizados (ECAs) y estudios cuasiexperimentales. En el grupo control se aceptaron: pacientes en lista de espera y pacientes que recibieran tratamiento usual u otra intervención neuropsicológica.

4. En medidas de resultados se aceptaron las relativas al funcionamiento cognitivo global, FE, atención o memoria.

5. Artículos publicados dentro del límite temporal (2008–2024).

Los criterios de exclusión fueron los siguientes:

1. Estudios con participantes que hayan padecido Ictus menores de 18 años.

2. Estudios realizados con sujetos sanos.

3. Intervenciones de RV no basadas en actividades diarias.

4. Revisiones o estudios no experimentales.

5. Estudios que no contuvieran medidas relativas al funcionamiento cognitivo.

6. Medidas cognitivas provenientes de escalas y cuestionarios autoadministrados.

7. Estudios que combinaran la intervención de RV con otro tipo de intervenciones.

8. Estudios publicados en idiomas distintos al inglés.

9. Artículos publicados fuera del rango temporal establecido (2008–2024).

### 2.2 Evaluación del Riesgo de Sesgo

Para evaluar el riesgo de sesgo de los estudios elegidos se utilizó la herramienta del Instituto Joanna Briggs (JBI), tanto de ECAs [28], como de estudios cuasiexperimentales [29]. Se emplearon listas de verificación para evaluar la claridad y el rigor de la estrategia de reclutamiento, la metodología de recopilación/análisis de datos, los resultados/medidas, las consideraciones éticas y la presentación de los hallazgos de los estudios. Ver Apéndice I (Ref. [22, 30, 31, 32, 33, 35]) y Apéndice II (Ref. [34, 36]).

### 2.3 Métodos de Análisis y Síntesis de la Información

Debido a la heterogeneidad de los estudios seleccionados, especialmente en los instrumentos de medida, los datos fueron sintetizados a nivel cualitativo, descartándose la elaboración de un metaanálisis.

La información obtenida de los estudios se codificó en una hoja de datos previamente diseñada para ello. Los datos obtenidos fueron: características generales (p. ej., autor y año), diseño del estudio, características de los participantes y de la intervención, comparadores y grupos controles, e instrumentos utilizados.

## 3. Resultados

### 3.1 Proceso de Selección de Artículos

**Fig. 1.**
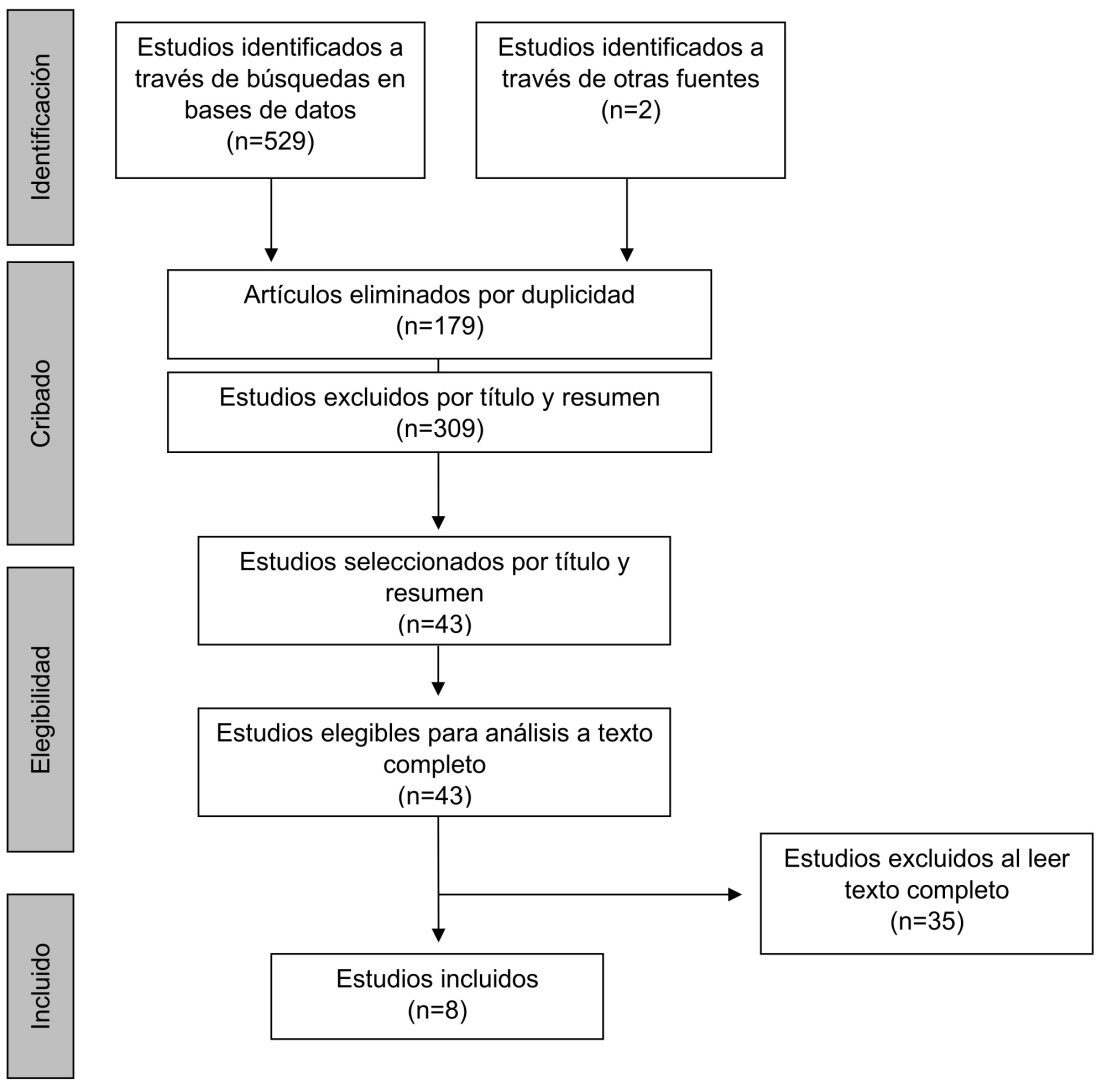
**Proceso de selección de los artículos según el diagrama PRISMA**. PRISMA, *Preferred Reporting Items for Systematic reviews and Meta-Analyses*.

El proceso de identificación, revisión y selección de estudios puede verse en la Fig. 1. En un primer momento se obtuvieron 531 artículos. Posteriormente, se eliminaron los duplicados (n = 179) utilizando el gestor de referencias *Refworks*. A continuación, se excluyeron artículos por título y *abstract* (n = 309). En último lugar, dos revisores analizaron los artículos restantes (n = 43) a texto completo de manera independiente. Se seleccionaron los artículos en los que hubo acuerdo de inclusión y en los que no, se debatió hasta llegar a un acuerdo. Finalmente se obtuvieron ocho estudios que cumplían con los requerimientos para formar parte de una revisión sistemática de la bibliografía. Las características descriptivas de los estudios incluidos pueden verse en la Tabla 3 (Ref. [22, 30, 31, 32, 33, 34, 35, 36]).

**Tabla 3.** **Estudios incluidos en la revisión**.

Autor y año	Título	Revista	Objetivo	Diseño	N
Specht* et al*. (2023) [22]	*Cognitive training with head-mounted display virtual reality in neurorehabilitation: pilot randomized controlled trial*	*JMR Serious Games*	Comparar efectividad entre RV de actividades diarias y entrenamiento cognitivo computarizado convencional	ECA	42
Chatterjee* et al*. (2022) [30]	*Immersive virtual reality for the cognitive rehabilitation of stroke survivors*	*IEEE Transactions on Neural Systems and Rehabilitation Engineering*	Determinar aceptación y seguridad del programa e identificar tendencias de mejora cognitivas o funcionalidad diaria post intervención y a los tres meses	ECA	40
Gamito* et al*. (2017) [33]	*Cognitive training on stroke patients via virtual reality-based serious games*	*Disability and Rehabilitation*	Evaluar efectividad de RV para el entrenamiento cognitivo en Ictus, comparándose con control en lista de espera	ECA	20
Faria* et al*. (2016) [31]	*Benefits of virtual reality based cognitive rehabilitation through simulated activities of daily living: a randomized controlled trial with stroke patients*	*Journal of NeuroEngineering and Rehabilitation*	Evaluar efectividad de intervención de RV comparado con tratamiento usual	ECA	18
Faria* et al*. (2020) [32]	*A comparison of two personalization and adaptive cognitive rehabilitation approaches: a randomized controlled trial with chronic stroke patients*	*Journal of NeuroEngineering and Rehabilitation*	Comparar entrenamiento en lápiz y papel personalizado con intervención de RV basada en AVD	ECA	36
De Luca* et al*. (2024) [35]	*Effects of virtual rehabilitation training on post-stroke executive and praxis skills and depression symptoms: a quasi-randomised clinical trial*	*Diagnostics*	Determinar la efectividad de la RV en la mejora de habilidades práxicas y la funcionalidad en individuos que padecieron Ictus severo.	Ensayo clínico cuasi-aleatorizado	20
Poulin* et al*. (2017) [34]	*Comparison of two cognitive interventions for adults experiencing executive dysfunction post-stroke: a pilot study*	*Disability and Rehabilitation*	Comparar viabilidad y eficacia de dos intervenciones para disfunción ejecutiva post ictus: (1) “Cognitive orientation to Daily occupational Performance”. (CO-OP) y (2) entrenamiento en FE mediante ordenador	Cuasi-experimental.	5
Oliveira* et al*. (2020) [36]	*Computerized cognitive training using virtual reality on everyday life activities for patients recovering from stroke*	*Disability and Rehabilitation: Assistive Technology*	Determinar la efectividad de la perspectiva ecológica de la RV, con demandas similares a las encontradas en la vida diaria, para la rehabilitación del Ictus.	Cuasi-experimental	30

ECA, ensayos clínicos aleatorizado; JMR, *Journal of Materials Research*; IEEE, *Institute of Electrical and Electronic Engineers*; AVD, actividades de la vida diaria.

### 3.2 Características de Los Estudios

Teniendo en cuenta todos los estudios incluidos, el total de las muestras fue de 211 personas, oscilando entre cinco y 42 por estudio. En la Tabla 4 (Ref. [22, 30, 31, 32, 33, 34, 35, 36]), se recopilan las características principales de los estudios.

**Tabla 4.** **Características de los estudios incluidos**.

Autor y año	Características de la muestra	Tipo de RV	Diseño	Características intervención	Instrumentos de medida
			Grupo 1 (G1): Intervención		
			Grupo 2 (G2): Control/comparador		
Specht* et al*. (2023) [22]	- Ictus subagudo	Inmersiva	G1: RV	18–25 sesiones (2–5 de 30 min/semana)	AKT, WMS-R, TMT, TL-D
	- Mini Mental >20	Programa: Teora mind	G2: Entrenamiento cognitivo computarizado convencional		
	- Índice Barthel (30–70)				
	- Edad media: 68				
	- Mujeres: 33%				
	- Alteración lenguaje: no especificado				
Chatterjee* et al*. (2022) [30]	- Ictus subagudo	Inmersiva	En adición a TAU:	10 sesiones (5/semana)	MOCA
	- Deterioro cognitivo (MOCA)	Programa: VIRTUE	G1: RV estratificado (severo y leve-moderado)		
	- Edad rango: 29–89				
	- Mujeres: 19		G2: RV falso		
	- Alteración lenguaje: no especificado				
Gamito* et al*. (2017) [33]	- Ictus (estadío no especificado)	No inmersiva	G1: RV	2–3 sesiones (60 min)/semana (en 4–6 semanas)	WMS-III, TTP, ROCF
	- Deterioro cognitivo (MOCA)		G2: lista de espera		
	- Edad media: 55				
	- Mujeres: 11				
	- Alteración lenguaje: no especificado				
Faria* et al*. (2016) [31]	- Ictus (hace una media de 5 meses)	No inmersiva	G1: RV	12 sesiones (20 min) (en 4–6 semanas)	ACE, TMT, WAIS-III (*Picture Arrangement*), MMSE
	- Con y sin deterioro cognitivo	Programa: Reh@City	G2: Rehabilitación convencional		
	- Edad media: 55,5				
	- Mujeres: 10				
	- Alteraciones lenguaje: se excluyen				
Faria* et al*. (2020) [32]	- Ictus (hace al menos 6 meses)	No inmersiva	G1: RV Reh@City 2.0.	12 sesiones	MOCA, TMT, WMS-III (*Verbal Paired Associates y Digit Span*),
	- Con y sin deterioro cognitivo (MOCA)		G2: Task Generator (entrenamiento adaptado en papel)		
	- Edad media: 62,07				WAIS (*Symbol Search and the Digit Symbol Coding)*
	- Mujeres: 16				
	- Alteración lenguaje: no especificado				
Poulin* et al*. (2017) [34]	- Ictus (desde hace 1,5 a 10 meses)	No inmersiva	G1: RV COMPUTER	16 sesiones (1 h) (2/semana en 8 semanas)	TMT, D-KEFS (CWIT), WAIS-IV (*Digit Span*)
	- Déficits en FE, sin deterioro en Mini Mental				
	- Edad: 34–79				
	- Mujeres: 2				
	- Alteraciones lenguaje: se excluyen				
Oliveira* et al*. (2020) [36]	- Ictus (desde hace 1-6 meses)	-no inmersiva	Un grupo	6–10 sesiones (30 min)	MOCA, FAB, WMS-I, CTT
	- Edad media: 60				
	- Mujeres: 12				
	- Alteraciones lenguaje: se excluyen				
De Luca* et al*. (2024) [35]	- Ictus (hace al menos 6 meses)	Inmersiva	G1: RV	24 sesiones	MMSE, FAB
	- Con deterioro cognitivo	Virtual reality rehabilitation System	G2: Tratamiento convencional	(3/semana de 60 min)	
	- Edad: 48,39 ± 14,77				
	- Mujeres: 50%				
	- Alteraciones lenguaje: se excluyen				

Nota. AKT, *alters-konzentrations-test*; WMS-R/WMS-III, escala de memoria de Wechsler-revisado/III; TMT, *trail making test*; FE, funciones ejecutivas; TAU, treatment as usual; TL-D, torre de londres-alemán; MOCA, evaluación cognitiva montreal; TTP, test de toulouse-pieron; ROCF, figura compleja de rey; ACE, *addenbrooke cognitive examination*; WAIS/III/IV, escala de inteligencia de Wechsler adultos; MMSE, *mini-mental state examination*; FAB, *frontal assessment battery*; CTT, *colour trails test*; D-KEFS, *Delis-Kaplan executive function system*; CWIT, *colour-word interference test*.

Del total de ocho estudios, cinco son ECAs [22, 30, 31, 32, 33], uno es un ensayo clínico parcialmente aleatorizado que se analizó como un estudio cuasiexperimental [34], otro es un ensayo clínico cuasi-aleatorizado [35] y el último es un estudio cuasi-experimental [36]. En los ECAs, como grupo control se utilizaron: entrenamiento cognitivo convencional/personalizado (n = 4) lista de espera (n = 1) y RV falsa (n = 1) (que presentaba un ejercicio simple repetidamente). Las intervenciones comprendieron una media de 14 sesiones de entre 20 a 60 minutos cada una, realizadas todas en presencia de un profesional. Tres estudios realizaron seguimiento (seg.) [30, 32, 34].

### 3.3 Características de la RV y Escenarios Representados

En cuanto al tipo de RV empleada, tres fueron inmersivas [22, 30, 35] y cinco no inmersivas [31, 32, 33, 34, 36]. Los inmersivos emplearon gafas de RV *Oculus* de la compañía *Meta* y los no inmersivos utilizaron ordenadores. Los escenarios de RV más empleados fueron: un supermercado [30, 31, 32] y una ciudad virtual [31, 32, 36]. El resto de los escenarios se distribuyeron en los estudios de una manera más equitativa, siendo: cocina, jardín, cuarto, baño, restaurantes, bancos, tiendas de ropa, farmacias, cruce de calle y transporte público. Todos los programas de RV empleados fueron creados para el área de rehabilitación cognitiva.

### 3.4 Instrumentos de Medida

En los instrumentos de medida utilizados se constató una gran variabilidad. Entre los instrumentos más empleados se encuentran el *Trail Making Test* (TMT) en cuatro estudios [22, 31, 32, 34], la Escala de Memoria de Wechsler en cuatro artículos (WMS-III) [22, 32, 33, 36]; el *Montreal Cognitive Assessment* (MOCA) en tres estudios [30, 32, 36] y la Escala de Inteligencia de Wechsler para Adultos (WAIS) en tres estudios: WAIS-III [31, 32] y el WAIS-IV [34]. El Test de la Figura compleja de Rey (RCFT) se empleó en una investigación [33], y el resto de los instrumentos se presentaron solo una vez en la totalidad de los estudios. Todos los instrumentos pueden visualizarse en la Tabla 4.

### 3.5 Medidas de Resultados

Los instrumentos que se tomaron como medidas de cada función cognitiva fueron los que los estudios propusieron para ellas. Los tamaños del efecto (TE, d, r) se indican siempre que los estudios los incluyan. En las Tabla 5 (Ref. [22, 30, 31, 32, 33, 35]) y Tabla 6 (Ref. [34, 36]) se reflejan los resultados principales.

**Tabla 5.** **Resultados ECAs según dominio cognitivo**.

Autor y año	Cognición global	Funciones ejecutivas		Atención	Memoria	Memoria de Trabajo	Memoria visual
Specht* et al*. (2023) [22]	-	=	Resolución	=	-	=	-
		Flexibilidad	problemas				
Chatterjee* et al*. (2022) [30]		-		-	-	-	-
Gamito* et al*. (2017) [33]	-	-			(WMS-III)	(WMS-III)	=
Faria* et al*. (2016) [31]		Intra (planificación)		C	-	-	-
Faria* et al*. (2020) [32]		= (ambos grupos mejora intragrupal)		C	= (episódica) (ambos grupos mejora intragrupal)	-	-
De Luca* et al*. (2024) [35]		Intra (FAB)		-	-	-	-

Nota. (Verde): Diferencias inter-grupales a favor de la intervención. (Rojo =): Ningún tipo de diferencias entre grupos. (Rojo C): Sólo diferencias intra-grupales en el grupo control. (Amarillo +/–): Ambiguo, contradicción entre tests. (Amarillo Intra): Ambiguo, sólo diferencias intra-grupales a favor de la intervención. (-): No evaluado. (FAB): Evalúa fluencia verbal, abstracción, planificación, flexibilidad cognitiva, impulsividad. (WMS-III): Evalúa memoria inmediata, memoria de trabajo y memoria demorada.

**Tabla 6.** **Resultados cuasiexperimentales según dominio cognitivo**.

Autor y año	Cognición global	Funciones ejecutivas		Atención	Memoria	Memoria de trabajo	Memoria visual y prospectiva
Poulin* et al*. (2017) [34]	-	Flexibilidad	Inhibición	-	-		-
Oliveira* et al*. (2020) [36]		(FAB)			(WMS-I)		

Nota. (Verde): Mejoría en los resultados. (Rojo): Similitud en los resultados. (-): No evaluado. “Resultados” hace referencia a medidas post-intervención. Se comparan con medidas pre-intervención para catalogar mejora o similitud. (FAB): Evalúa fluencia verbal, abstracción, planificación, flexibilidad cognitiva, impulsividad. (WMS-I): Evalúa memoria inmediata, memoria de trabajo y memoria demorada.

### 3.6 Funcionamiento Cognitivo Global

Tres ECAs [30, 31, 32], un ensayo clínico cuasi-aleatorizado [35] y un estudio cuasi-experimental [36] valoraron el funcionamiento cognitivo global y encontraron diferencias intergrupales significativas (comparación intervención-control) a favor del grupo intervención. Faria* et al*. ([32], 2020) las encuentran en el MOCA (U = 65,00;* p* = 0,020; r = 0,41). Chatterjee* et al*. [30], las indican en el grupo de deterioro cognitivo severo en el MOCA (Media [rango] Grupo severo: 8 [3–17] y Control: 0 [(–4)–10]). Faria* et al*. ([31], 2016) las encuentra en el ACE (U = 13,500; *p* = 0,014; r = 0,56) y en el *mini-mental state examination* (MMSE) (U = 18,000; *p* = 0,050; r = 0,47). De Luca* et al*. [35] las encontraron en el MMSE (2,13 a 4,67; *p *= 0,002). Por último, el estudio cuasi experimental, encontró diferencias intragrupales significativas en el MOCA [t(29) = –4,746; d = 0,54; *p* < 0,001] [36].

### 3.7 Funciones Ejecutivas

Seis estudios midieron las FE [22, 31, 32, 34, 35, 36]. Un ECA encontró diferencias intergrupales a favor del grupo intervención en el test torre de londres-alemán (TL-D) y ningún tipo de diferencia significativa en la ejecución del test TMT [22]. Las diferencias intragrupales (comparación pre-post dentro de cada grupo) se detectaron en: Faria* et al*. ([31], 2016) en *picture arrangement* de la prueba WAIS en el grupo intervención (W = 21,000; *p* = 0,026; r = 0,74); Faria* et al*. ([32], 2020), en el grupo intervención (W = 87,00; *p* = 0,030; r = 0,58) y en el control (seg.) (W = 101,00; *p* = 0,019; r = 0,55), y De Luca* et al*. [35] en el grupo intervención en el test *frontal assessment battery* (FAB) (0,96 a 1,63; *p* = 0,002).

En cuanto a los estudios cuasi-experimentales, Poulin* et al*. [34]encontraron TE grandes para *colour-word interference test* (CWIT) inhibición (post = –0,6 y seg. = –0,9) y Oliveira* et al*. [36], en el test FAB [t(28) = –2,801; d = 0,40; *p <* 0,01].

### 3.8 Atención

Cinco estudios evaluaron la atención [22, 31, 32, 33, 36]. Gamito* et al*. [33] encontraron diferencias intergrupales significativas a favor del grupo intervención en el dominio de eficiencia de trabajo en atención sostenida del *Test Toulouse-Pieron* (TPT) (F = 4,719; *p* = 0,044). En cuanto a las diferencias intragrupales, dos estudios encontraron mejoras en el grupo control: Un estudio, en el TMT-A (W = 18,00;* p* = 0,010; r = 0,61) [32] y el otro estudio en el TMT B (W = 0,000; *p* = 0,039; r = 0,69) [31]. Specht* et al*. [22], no encontraron diferencias de ningún tipo. El estudio cuasi-experimental, encontró mejoras en el test *colour trails test* (CTT) [t(12) = –6,199; d = 0,70; *p* < 0,001] [36].

### 3.9 Memoria

Tres estudios evaluaron la memoria [32, 33, 36]. Gamito* et al*. [33] indicaron una diferencia intergrupal en la WMS (F = 4,745; *p *= 0,043). Faria* et al*. ([32], 2020) indicaron mejoras intragrupales significativas en ambos grupos en el *verbal paired associates* (WMS-III); el grupo intervención en retención (W = 36,00; *p* = 0,012; r = 0,67) y reconocimiento (W = 21,00; *p* = 0,027; r = 59), y el control, en retención (post: W = 118,0; *p* = 0,009; r = 0,61; Seg.: W = 95,00; *p* = 0,006; r = 0,65); y el estudio cuasi-experimental encontró mejoras significativas en la WMS [t(25) = –3,297; d = 0,39; *p* < 0,01] [36].

Respecto a la memoria de trabajo: un ECA indicó una tendencia de mejora no significativa en Dígitos (WMS-R) (*p* = 0,18) [22] y un estudio cuasiexperimental observó efectos de tamaño grande en el *digit span* (*back.:* post = 1,2 y seg. = 1,6 y *seq.:* post = 1,3 y seg. = 0,3) [34]. En cuanto a la memoria visual, Gamito* et al*. [33] no indicaron efectos significativos.

### 3.10 Seguimiento

Tres estudios realizaron seguimiento dos-tres meses después de la finalización de la intervención. Dos estudios encontraron que las mejoras se mantenían [30, 34], y el otro estudio indicó que solo se mantuvieron en el grupo control [32].

## 4. Discusión

Esta revisión se realizó con el objetivo principal de analizar la evidencia disponible sobre el uso de la RV basada en actividades diarias para la rehabilitación cognitiva de personas con Ictus mayores de 18 años. En concreto, se analizó el impacto en el funcionamiento cognitivo global, las FE, atención y memoria. Se revisaron ocho artículos (seis ECAs y dos estudios cuasiexperimentales), identificándose una mayor proporción de resultados positivos y una cantidad considerable de resultados negativos. Si profundizamos en ellos, se observa que la mayoría de los resultados positivos solo se dan en el funcionamiento cognitivo global. La hipótesis formulada para este objetivo principal planteaba que esta intervención en RV sería beneficiosa tanto en esta área como en funciones cognitivas específicas debido a que en las recomendaciones actuales en rehabilitación cognitiva del DCA el enfoque es especialmente contextualizado [9, 10, 11, 12]. Además, debe tenerse en cuenta que si sólo se consideran los ECAs, los resultados positivos y negativos se igualan. En vista de los resultados, esta primera hipótesis debe rechazarse parcialmente. A pesar de las diferencias de nuestro trabajo con la revisión de Faria* et al*. [26], estos resultados van en línea con sus hallazgos, que indicaban falta de conclusiones robustas.

En las revisiones sobre intervención cognitiva en DCA con RV sin diferenciación de tareas presentadas, los resultados son mayormente positivos [24, 37, 38]. No obstante, una revisión no encuentra evidencia de su efectividad [39] y otra revisión duda sobre si las mejoras cognitivas se transfieren a las AVD [37]. En ambas, se alude a las limitaciones de los estudios incluidos como explicación para sus resultados. En la presente revisión también se detectaron esas limitaciones, sin embargo, las dudas sobre la transferencia de eficacia desde medidas cognitivas a AVD y viceversa es un punto interesante que también supone una posible hipótesis explicativa para nuestros resultados.

A excepción de la revisión de Faria* et al*. [26], no consta que existan otras revisiones sobre RV basada exclusivamente en actividades diarias en DCA. En el área de deterioro cognitivo leve y enfermedad de Alzheimer, existe una revisión sobre esta intervención que presenta resultados positivos [40]. Sin embargo, esta toma la ejecución de AVD como medida de resultado, a diferencia de nuestra revisión, que sólo incluyó medidas del funcionamiento cognitivo. A pesar de las diferencias entre las poblaciones estudiadas, esto podría explicar los resultados obtenidos en la presente revisión, ya que es esperable que una intervención de RV basada en actividades diarias manifieste su efectividad en medidas de AVD y no necesariamente en medidas cognitivas. Aun así, debe tenerse en cuenta que el paradigma de rehabilitación cognitiva contextualizada indica que el entrenamiento en tareas contextualizadas podría conllevar la reducción de la alteración cognitiva en sí [8]. Si bien esta reducción en la alteración podría reflejarse en las medidas de funciones cognitivas, no se especifica tras cuanto tiempo de intervención se conseguiría. Por lo tanto, también podría haber ocurrido que, en caso de que el impacto solo se haya dado en las AVD, no hubiese transcurrido el tiempo suficiente para ver esa transferencia a las medidas cognitivas. Para poder llegar a una conclusión sobre este aspecto, sería necesario contar con más estudios que realicen seguimiento para detectar posibles transferencias. Además, es importante que los estudios incluyan medidas de AVD; especialmente las relacionadas con el funcionamiento cognitivo. Si bien es verdad que en esta revisión no se establecieron las medidas de AVD como un criterio de inclusión, en la mayor parte de los estudios revisados que las evaluaron, las AVD no estuvieron específicamente relacionadas con el funcionamiento cognitivo [30, 31, 32, 34]. Una hipótesis explicativa de este hecho puede ser la falta de instrumentos que evalúen las áreas de las AVD mayormente afectadas por un funcionamiento cognitivo deficitario, ya que sigue siendo el funcionamiento motor el más estudiado y valorado en relación a ellas.

Por otro lado, el paradigma de rehabilitación contextualizada y las recomendaciones más recientes incluyen técnicas de intervención eficaces, como estrategias cognitivas [8, 9, 10, 11]. En la actual revisión se descartaron los estudios que combinaban intervenciones debido a la variedad de combinaciones existentes, excluyéndose así, el único estudio identificado que incluía entrenamiento en estrategias cognitivas [41]. Por tanto, los estudios revisados solo evaluaron intervención con RV. Esto también podría explicar la falta de resultados satisfactorios. El uso de la RV basada en actividades diarias como escenario de entrenamiento de intervenciones cognitivas eficaces, que además cuente con la guía del terapeuta, sería la manera más interesante de implementar esta tecnología en rehabilitación por lo que se debería estudiar su efectividad.

De los objetivos específicos, el primero y el segundo pretendían analizar los cambios que se daban en el funcionamiento cognitivo tras la intervención con RV basada en actividades diarias y si existía una función cognitiva que se viera más beneficiada que las demás. Se había hipotetizado que las FE obtendrían mejores resultados debido a que es el único dominio donde la INCOG 2.0 recomienda la RV con un nivel de evidencia A (si bien las tareas de RV estudiadas para llegar a esa conclusión fueron variadas y no exclusivamente basadas en actividades diarias) [9]. Esta hipótesis se rechaza, ya que los mejores resultados se dieron en el funcionamiento cognitivo global. Además de las explicaciones otorgadas al discutir la primera hipótesis, otra explicación puede ser que los estudios utilizaron más instrumentos para evaluar FE (obteniendo disparidad en los resultados), que para evaluar el funcionamiento cognitivo global (empleando instrumentos de *screening)*. Sin embargo, también podría deberse a que los cambios logrados fueran pequeños y sólo se detectase su efecto combinado en una evaluación global. Respecto al tercer objetivo, se analizaron los instrumentos de medidas utilizados y se detectó poca uniformidad [24], lo que conlleva la dificultad de comparación de resultados. Para el cuarto objetivo, relativo a la existencia de medidas de seguimiento, estas se detectaron en tres estudios dos-tres meses después [30, 32, 34]. La falta de seguimiento no permite saber si los cambios se mantienen a largo plazo y dificulta establecer las características de un programa de intervención efectivo [42].

Las limitaciones de los estudios incluidos también pueden explicar los resultados. La limitación principal es el pequeño tamaño de las muestras y escasez de ECAs. Esto podría deberse a la poca accesibilidad a dispositivos y programas de RV, especialmente a los de nivel inmersivo. La falta de accesibilidad a estos últimos podría ser debida a que los dispositivos inmersivos son más costosos y no se ha podido determinar si son más exitosos en intervención cognitiva que los dispositivos menos inmersivos [42]. Sin embargo, es justamente la falta de estudios de alta calidad la que dificulta la obtención de evidencia sobre su efectividad [39, 42]. Otra razón podría ser que la tecnología de RV inmersiva puede causar sensaciones corporales no deseadas (p. ej., nauseas), englobadas bajo el término “ciberenfermedad” [43]. La ciberenfermedad podría generar una reticencia en el uso de dispositivos inmersivos, si bien en los estudios revisados hubo alta aceptación. El hecho de que la mayor parte de los estudios incluidos utilicen tecnología no inmersiva puede estar afectando a las conclusiones extraídas del uso de RV en esta área, por lo que realizar estudios que discriminen según el nivel de inmersión se vuelve un aspecto necesario. Paralelamente, dado que el área cognitiva no es el principal foco de la intervención en RV, existen pocos programas de entrenamiento enfocados en ella [44], siendo aún menos los basados en actividades diarias. El estudio más profuso y estructurado de esta tecnología nos ayudaría a conocer el verdadero valor ecológico de la misma, ya que existen otras variables interesantes que podrían aumentar ese valor. Por ejemplo, el uso de material inmersivo real (videos e imágenes del mundo real, no digitales) y tecnología semi-inmersiva combinados con material real y manipulable [45]. Si bien en esta revisión sólo se aceptaron programas digitales y se excluyeron combinaciones de esta tecnología con otros materiales, sería interesante estudiar y revisar la efectividad de la combinación de distintas variables que podrían aumentar el valor ecológico de este tipo de intervención. Entre otras limitaciones de los estudios se encuentran: la falta de medidas de resultados relativas al impacto del funcionamiento cognitivo en las AVD, siendo solo un estudio el que las incluyen [34] y también, la diversidad de instrumentos de medida empleados y la falta de concordancia entre los estudios a la hora de establecer las funciones cognitivas que evalúa cada uno.

Entre las limitaciones de la presente revisión se encuentra, principalmente, la inclusión de estudios cuasiexperimentales debido a la escasez de ECAs. Además, debido a muestras pequeñas algunos estudios se centraron principalmente en la aceptabilidad de la intervención y no en su efectividad [30, 34]. También se detectaron grupos control diversos (p. ej., lista de espera, comparación con intervención cognitiva eficaz), lo que podría haber generado distorsión en los hallazgos de diferencias intergrupales. Además, se acepta una posible pérdida de información: la búsqueda se realizó en tres bases de datos y en idioma inglés, la cantidad de términos de búsqueda fue pequeña debido a la exclusión de términos que generaban ruido documental, y se excluyeron algunos dominios cognitivos debido a la especificidad de su condición e intervención (negligencia espacial, afasias y orientación espacial). En esta línea, la inclusión de términos relativos a AVD como medidas de resultados habría sido interesante y acorde al objetivo de nuestro trabajo. Sin embargo, tuvo que descartarse debido al ruido documental que generaba su incorporación.

En cuanto a futuros estudios, en línea con las limitaciones de los estudios planteadas, se hace clara la necesidad de establecer un consenso sobre las variables metodológicas y criterios mínimos necesarios para la realización de estudios en este ámbito. Dicho consenso podría establecerse siguiendo el método Delphi [46], es decir, a través de un grupo de trabajo de expertos para la conformación de guías a seguir. Así, se facilitaría la realización de estudios de alto valor metodológico y por tanto, la obtención de conclusiones robustas y de calidad. Mas allá, de los esfuerzos por superar cuestiones metodológicas que limitan el alcance de esta intervención, sería interesante realizar estudios que comparen la efectividad de tareas descontextualizadas frente a tareas basadas en actividades diarias, ambas presentadas en RV. Además, valorar el impacto de la intervención en las AVD con especial foco en su afectación debida al funcionamiento cognitivo. Por último, explorar el uso de esta intervención combinada con otras técnicas rehabilitadoras, como por ejemplo, estrategias cognitivas.

## 5. Conclusiones

El principal objetivo de este trabajo fue analizar la evidencia disponible sobre el uso de la RV basada en actividades diarias en la rehabilitación cognitiva de personas con Ictus mayores de 18 años. Mediante la revisión de ocho artículos, la conclusión principal es que la evidencia encontrada es ambigua, lo que da lugar a un rechazo parcial de la hipótesis principal, debido a que los resultados positivos se encuentran mayoritariamente en el funcionamiento cognitivo global pero hay escasez de ellos en funciones cognitivas específicas, especialmente en los ECAs. También, existen dudas sobre si alguna función cognitiva pueda verse más beneficiada que las demás. Se destaca que las medidas de resultados empleadas y las limitaciones de los estudios, como la falta de ECAs con muestras de mayor tamaño, pudieron haber influido notablemente en estos resultados. Subsanar estas limitaciones permitiría llegar a conclusiones más robustas. Además, sería especialmente importante la comparación de la efectividad de la intervención en RV basada en actividades diarias frente a intervenciones de RV basadas en tareas descontextualizadas y también el estudio de la efectividad del uso de RV basada en actividades diarias como escenario de entrenamiento virtual de distintas técnicas y estrategias de rehabilitación cognitiva.

## Figures and Tables

**Table:** Apéndice I. Valoración de riesgo de sesgo (ECAs).

	¿Verdadera aleatorización en asignación de grupos?	¿Se ocultó la asignación a los grupos?	¿Grupos similares al inicio?	¿Participantes cegados a la asignación al tratamiento?	¿Administradores del tratamiento cegados a la asignación?	¿Grupos tratados igual a excepción de la intervención?	¿Evaluadores de resultados cegados a la asignación del tratamiento?	¿Resultados medidos de igual manera en los grupos?	¿Resultados medidos de manera fiable?	¿Seguimiento completo y si no, las diferencias entre grupos se describieron y analizaron?	¿Participantes analizados en sus grupos correspondientes?	¿Análisis estadístico apropiado?	¿Diseño del ensayo apropiado y cualquier desviación contabilizada en la realización y análisis?
Specht *et al*. (2023) [22]	●	●	●	●	●	●	●	●	●	●	●	●	●
Chatterjee *et al*. (2022) [30]	●	●	●	●	●	●	●	●	●	●	●	●	●
Gamito *et al*. (2017) [33]	●	●	●	●	●	●	●	●	●	●	●	●	●
Faria *et al*. (2016) [31]	●	●	●	●	●	●	●	●	●	●	●	●	●
Faria *et al*. (2020) [32]	●	●	●	●	●	●	●	●	●	●	●	●	●
De Luca *et al*. (2024) [35]	●	●	●	●	●	●	●	●	●	●	●	●	●

Nota. Sí (verde). No (rojo). Dudoso (amarillo). No aplica (-).

**Table:** Apéndice II. Valoración de riesgo de sesgo (cuasiexperimentales).

	¿Está clara la causa y el efecto?	¿Participantes similares en cualquier comparación?	¿Participantes en cualquier comparación recibieron trato similar a excepción de la intervención?	¿Hubo grupo control?	¿Se realizaron varias medidas de los resultados pre y post intervención?	¿Seguimiento completo y si no, las diferencias entre los grupos fueron descritas y analizadas?	¿Resultados en cualquier comparación medidos de igual manera?	¿Resultados medidos de manera fiable?	¿Análisis estadístico apropiado?
Poulin *et al*. (2017) [34]	●	●	●	●	●	●	●	●	●
Oliveira *et al*. (2020) [36]	●	-	-	●	●	●	-	●	●

Nota. Sí (verde). No (rojo). No aplica (-).

## Data Availability

Todos los puntos de datos generados o analizados durante este estudio se incluyen en este artículo y no hay otros datos subyacentes necesarios para reproducir los resultados.

## Contribuciones de los Autores

Conceptualización del estudio, diseño metodológico (FSL-I y DÍ-F), redacción del manuscrito, recolección y análisis de datos, interpretación de resultados (FSL-I). Supervisión, revisión crítica del manuscrito, aprobación final (DÍ-F). Todos los autores leyeron y aprobaron el manuscrito final. Todos los autores han participado lo suficiente en el trabajo y han acordado ser responsables de todos los aspectos del mismo.

## Aprobación Ética y Consentimiento Informado

No aplicable.

## Agradecimientos

No aplicable.

## Financiación

Esta investigación no recibió financiación externa.

## Material Suplementario

El material suplementario asociado con este artículo se puede encontrar, en la versión en línea, en https://doi.org/10.31083/RN37507.

## Apéndice

See Apéndices I,II.
